# Supplementary figures and images for: Genome-Wide Characterization of the ANN Gene Family in Corydalis saxicola Bunting and the Role of CsANN1 in Dehydrocavidine Biosynthesis
Source: Plants (Basel). 2025 Jun 27;14(13):1974. doi: 10.3390/plants14131974 (PMC12252069; doi:10.3390/plants14131974)

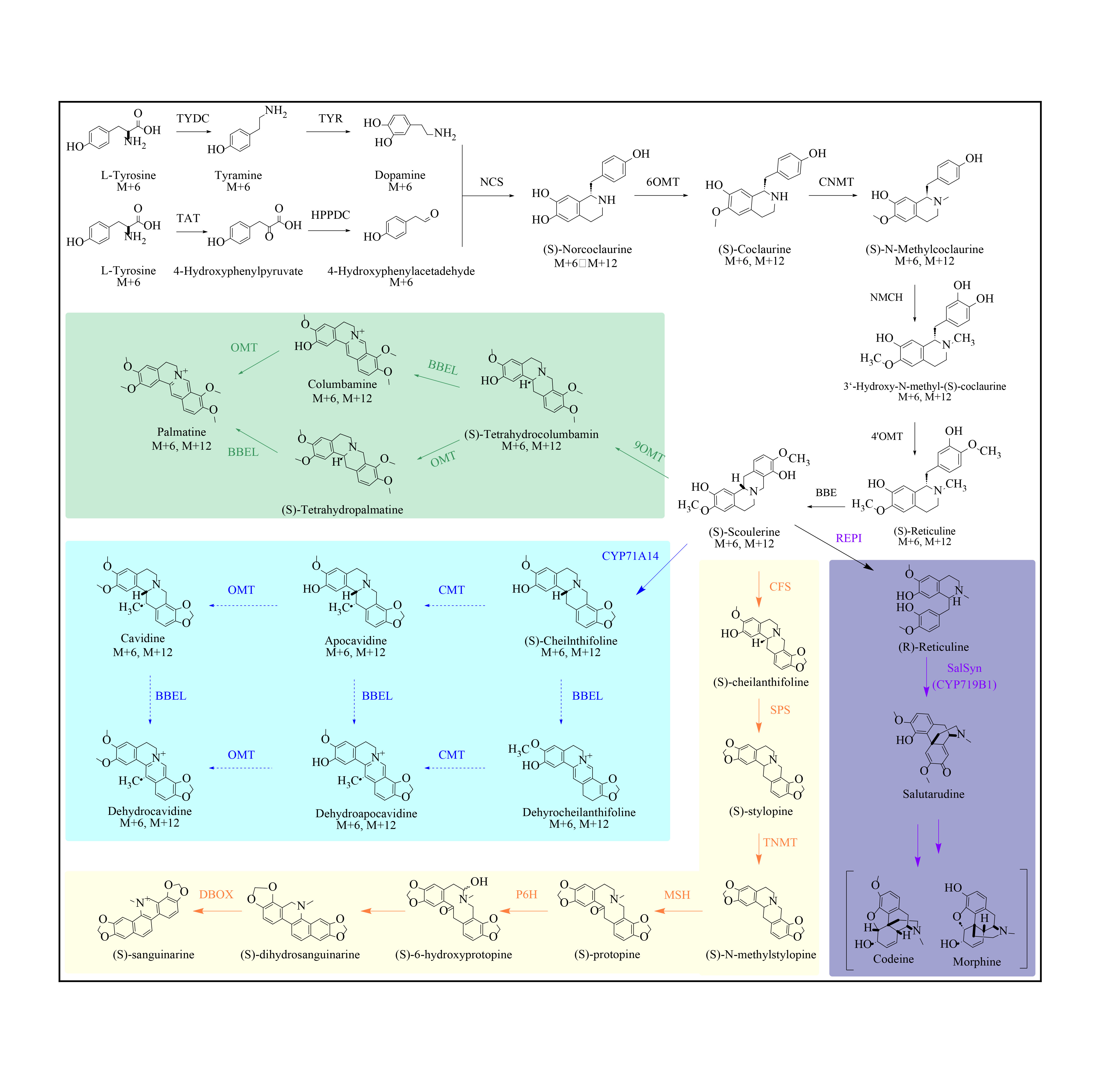

Supplement: Supplementary file 1 [file plants-14-01974-s001.zip › Figure S1.jpg]

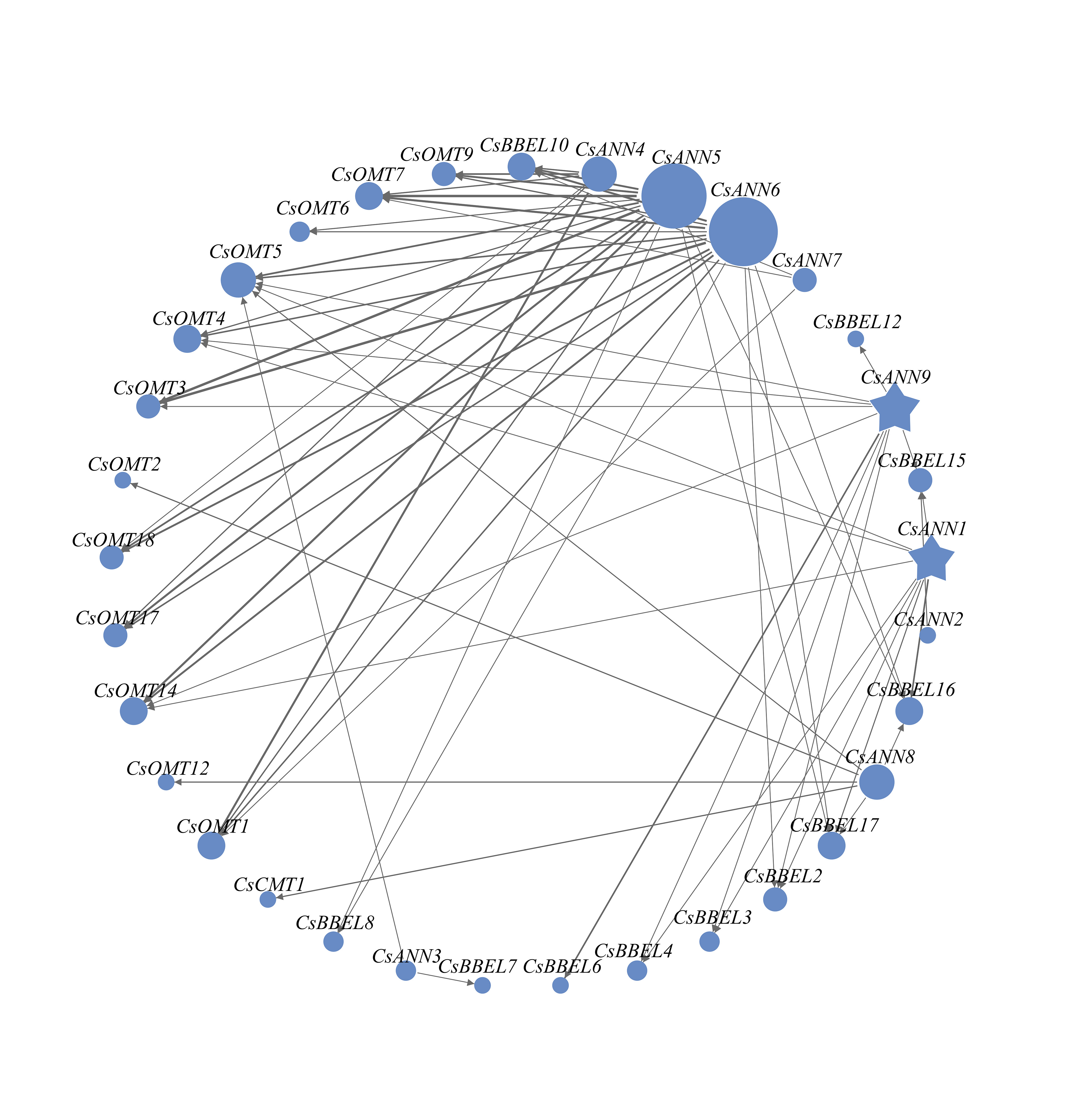

Supplement: Supplementary file 1 [file plants-14-01974-s001.zip › Figure S2.jpg]

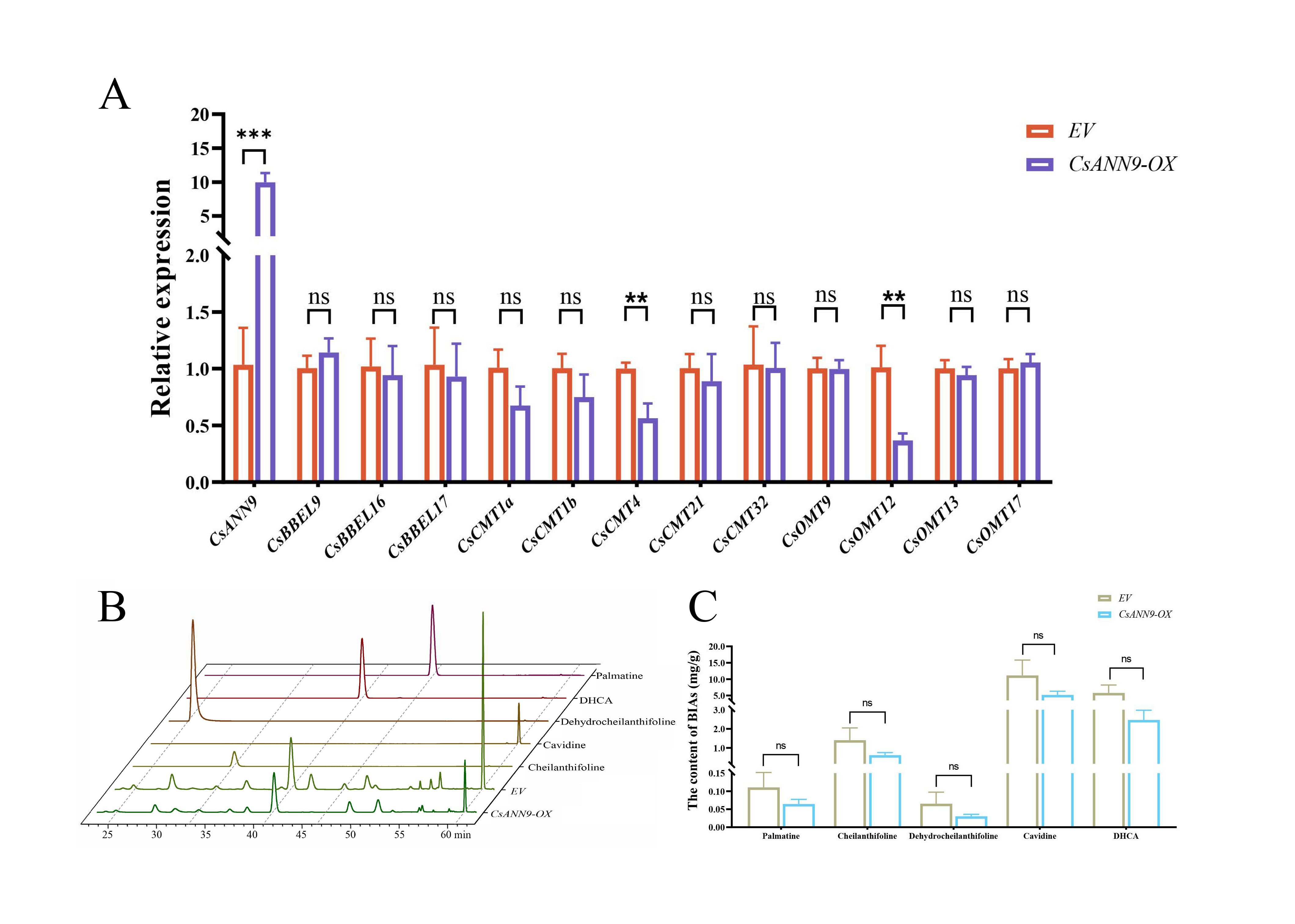

Supplement: Supplementary file 1 [file plants-14-01974-s001.zip › Figure S3.jpg]

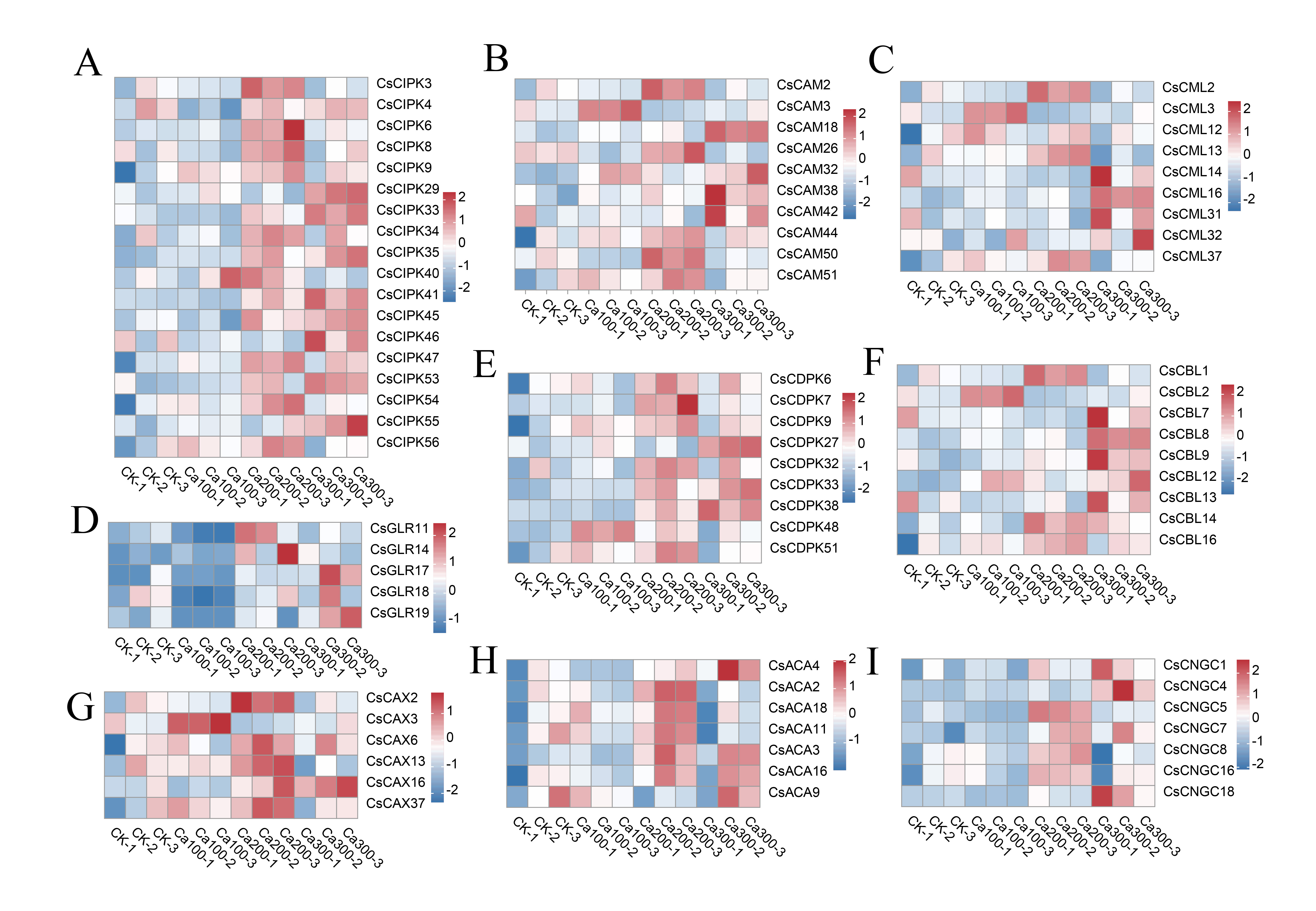

Supplement: Supplementary file 1 [file plants-14-01974-s001.zip › Figure S4.jpg]

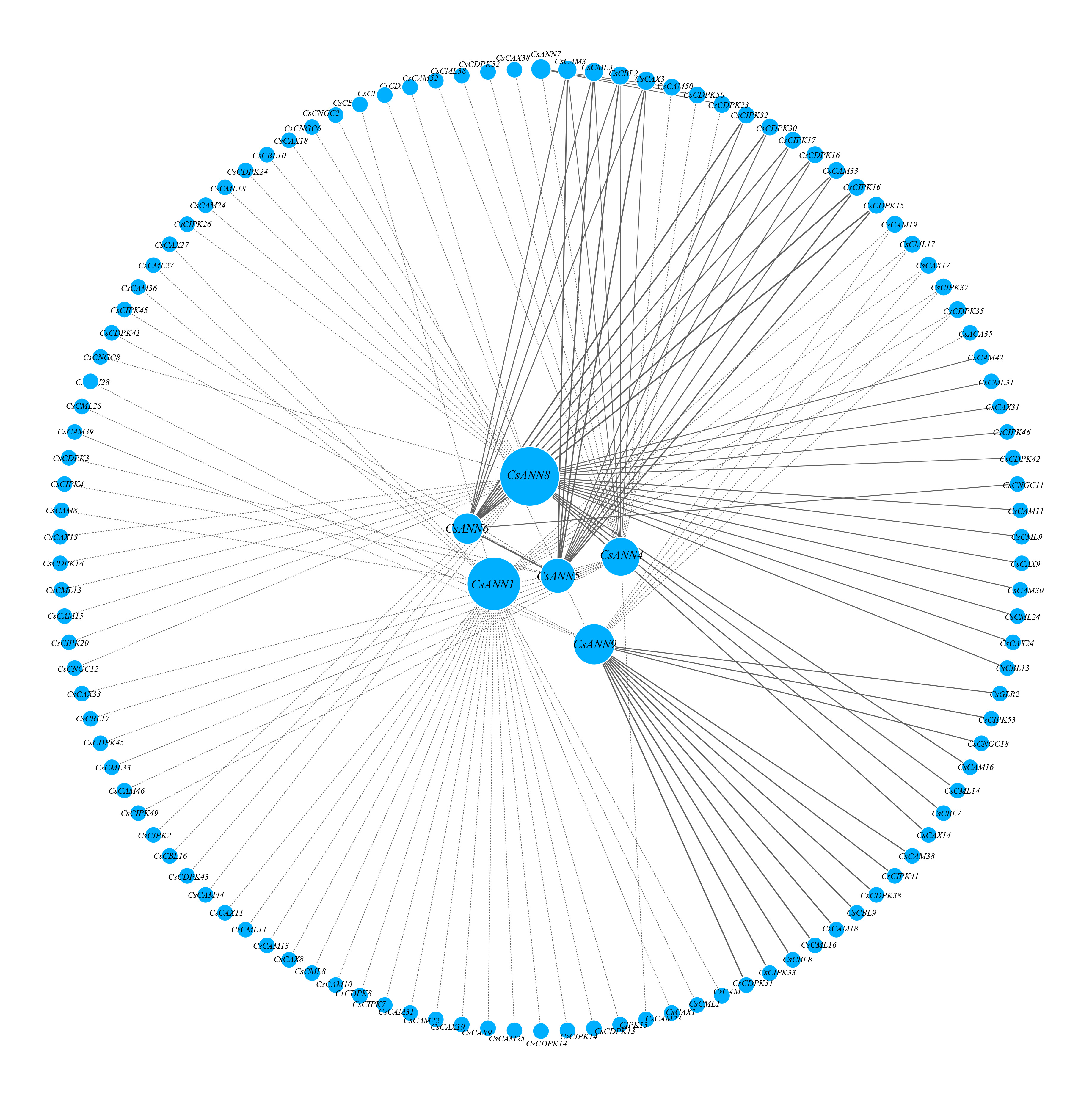

Supplement: Supplementary file 1 [file plants-14-01974-s001.zip › Figure S5.jpg]
